# Protocol: Understanding the Content, Context, and Impact of Far‐Right Extremist Propaganda Disseminated Online: A Systematic Review

**DOI:** 10.1002/cl2.70076

**Published:** 2025-11-14

**Authors:** Mia Doolan, Katie Cox, Kiran M. Sarma

**Affiliations:** ^1^ School of Psychology University of Galway Galway Ireland

## Abstract

This is the protocol for a Campbell Systematic Review. This review will address two aims: (1) A qualitative synthesis of literature on the composition of online far right propaganda, and (2) A quantitative synthesis of literature examining the impact of exposure to online far‐right propaganda on audiences. These syntheses will be guided by the following specific objectives: (i) What is the content (i.e. themes) of online far‐right propaganda, and how does this differ across ideological subgroups? (ii) What is the structure of online far‐right propaganda, and how does this differ across ideological subgroups? (iii) What is the context of these messages (i.e., where, when and by whom were they posted?) (iv) What impact does exposure to online far‐right propaganda have on audiences with reference to the radicalisation of opinion and/or action?

## 1 Background

### 1.1 The Problem, Condition or Issue

The mobilisation of far‐right extremist groups is regarded as the fastest‐growing and most prominent domestic security threat facing several UN member states (UN Secretary General 2022). According to the Right‐Wing Terrorism and Violence Data set compiled by the Centre for Research on Extremism (C‐REX), there were 93 attacks in Europe in 2022, 4 of which resulted in fatalities (Aasland et al. 2023). This violence has coincided with the increasing popularity of far‐right politics in many societies, as the far‐right moves from the margins to mainstream politics (Völker and Saldivia Gonzatti 2024). These developments underscore the need for Preventing and Countering Violent Extremism (P/CVE) stakeholders to investigate the mechanisms underlying the spread of far‐right influence, where it may lead to violence, and develop interventions to mitigate the harmful effects of far‐right messaging.

As the growth of so‐called ‘far‐right influence’ continues, debate has emerged about the parameters of this term – what constitutes a ‘far‐right’ group or individual? Many different labels are employed throughout the literature to describe extremist groups on the right side of the political spectrum, including ‘extreme right’, ‘hard right’, ‘ultra‐right’ and ‘radical right’ (Stojarova 2016, 8–19). These groups remain an ‘ideo‐politically variegated phenomenon’, but nonetheless, form part of a connected network of extreme right‐wing influence (Anievas and Saull 2022, 715). For the purposes of this review, we use the umbrella term of ‘far‐right’ to encompass these groups (Pirro 2022). Our operational definition of the term ‘far‐right’ will adopt the following definition put forward by the European Centre for Populism Studies:

‘[a term used] to describe the historical experiences of fascism and Nazism, it today includes neo‐fascism, neo‐Nazism, Third Position, the alt‐right, white nationalism and other ideologies or organisations that feature ultranationalist, chauvinist, xenophobic, theocratic, racist, homophobic, anti‐communist, or reactionary views.’ (European Centre for Populism Studies 2019, para. 1).

To understand the growth and mobilisation of far‐right groups, P/CVE scholars analyse far‐right communications to investigate how they attempt to influence their target audience. Such research seeks to understand the social and psychological processes by which one becomes persuaded to adopt far‐right beliefs, and possibly endorse, support, or take part in subsequent action. This move from attitudinal, emotional or behavioural neutrality to attitudinal, emotional or behavioural support for violent extremism is conceptualised by P/CVE scholars as violent radicalisation.

For this review, we adopt the two‐pyramid model of radicalisation proposed by McCauley and Moskalenko (2017), which distinguishes between radicalisation of opinion and radicalisation of action. Radicalisation of opinion (or cognitive radicalisation) refers to attitudinal and emotional shifts from neutrality to justification of violence, to personal moral obligation to commit violence on behalf of a cause. Radicalisation of action (or behavioural radicalisation) refers to the shift in behaviour from one of inertia to radical action or potentially acts of violent extremism. The distinction between attitudinal change and behavioural change is important, as the P/CVE literature demonstrates that ‘radical opinions are neither necessary nor sufficient for terrorist violence’ (McCauley and Moskalenko 2017, 213). McCauley and Moskalenko's model was chosen as a framework for this review as it distinguishes between the psychological processes involved in radicalisation to extremist beliefs and radicalisation to violent extremism. Furthermore, the model takes into account individual emotional experiences, as well as wider environmental factors involved in radicalisation, both of which are relevant to this review (Bognár and Farkas 2025).

Of particular importance to this study is the role of far‐right propagandistic messaging in the process of radicalisation. While debate exists as to the definition of propaganda, this review adopts the framework proposed by Jowett and O'Donnell (2012), which identifies propaganda as a ‘deliberate, systematic attempt to shape perceptions, manipulate cognitions, and direct behaviour to achieve a response that furthers the desired intent of the propagandist’ (p. 4).

Propaganda plays an important role in political persuasion, with scholars identifying so‐called ‘propaganda‐effects’; specific techniques utilised to enhance the ‘potency’ of a persuasive effort, and induce changes in an audience (Perloff 2013). As Jowett and O'Donnell (2012) note, these changes can be cognitive, behavioural, perceptual, or a combination of the three.

An important element of extremist propaganda is the use of narratives (Braddock and Dillard 2016; Carthy et al. 2020; Walsh and Gansewig 2024). A narrative can be understood as a ‘symbolic representation of events’, containing chronology (organisation of events in time, i.e., beginning, middle and end), and causality (the structuring of story elements into an organised framework that establishes relationships between the elements and allows for causal inference; Bilandzic and Busselle 2012). Social science research has evidenced how narratives operate by transporting and immersing the audience in the story within the narrative, a process thought to facilitate persuasion (Brechman and Purvis 2015). Another mechanism of narrative persuasion is identification; the extent to which an individual adopts the perspective of characters present within a narrative. Identification allows an individual to vicariously adopt the beliefs and goals of characters, facilitating emotional immersion within the events of the story (Hoeken et al. 2016). Successful identification with a character also facilitates parasocial interaction, another important mechanism of narrative persuasion. Parasocial interaction refers to the pseudo‐relationship formed between the audience and characters within a narrative (Moyer‐Gusé 2008). Similar to that of a real relationship, parasocial interaction facilitates attachment and a greater emotional investment in the decisions made and events experienced by characters within a story (Shen et al. 2018).

Extremist narratives operate via these mechanisms, with the audience immersed in the characters and events created by the propagandist (Carthy et al. 2020). These narratives often contain emotional appeals, including those targeting fear and empathy, to elicit desired responses. Extremist narratives can be created around themes including persecution, conspiracy, or dystopia (Berger 2018). Examples include conspiracy theory narratives about a ‘global cabal’ of powerful Jews controlling society, an anti‐Semitic narrative often circulated in far‐right spaces (Hermansson et al. 2020). Combining the persuasive power of propaganda effects and the persuasive power of narrative storytelling, extremist narratives immerse the audience in the worldview of the propagandist.

The internet serves as an important tool in disseminating extremist narratives and has been used by the far‐right dating back to the 1990s (Conway et al. 2023; Conway et al. 2019). Modern social media platforms and internet technologies can now facilitate quick and cost‐effective dissemination of extremist propaganda across the globe, and scholars have documented the role of an extensive online ecosystem in the spread of far‐right propaganda (Baele et al. 2020; Badawy and Ferrara 2018; Rieger et al. 2019). As the expansion of this space and the reach of far‐right actors grows, social scientists and communication scholars are striving to identify the persuasive techniques which underly the persuasive potency of far‐right propaganda (Capecchi 2021; Simpson and Druxes 2015).

Analysis of far‐right propaganda has shed light on the themes and narratives that far‐right groups deploy in their messaging. These include use of fantasy discourse and narratives involving heroism in appeals to young audiences (Nilan 2021), race propaganda (conspiracy theories of ‘white victimisation’ and ‘white genocide’; Castle et al. 2018), visual ‘meme’ humour (Kelly 2023), and narratives of martyrdom involving past perpetrators of mass violence (e.g., Robert Bowers and Anders Breivik; Am and Weimann 2020). Analysis of manifestos and live‐streams generated by perpetrators of far‐right terrorism evidence the ‘contagious’ effect of far‐right propaganda and narratives, as individual authors often reference one another directly, replicate the content and structure of other manifestos, and use similar themes and language to announce planned attacks (Kupper et al. 2022).

This review aims to synthesise this literature on the content, structure, and context of far‐right propaganda to develop an understanding of how it is composed. Additionally, we will review findings related to the impact of far‐right propaganda on audiences, to develop an understanding of how exposure to this messaging can influence cognitive and behavioural outcomes, for example, voting behaviours (Malet 2024), and implicit attitudes (Matthes and Schmuck 2015). We will categorise this impact as either: (a) radicalisation of opinion, (b) radicalisation of action, or both, with reference to the two‐pyramid model of radicalisation (McCauley and Moskalenko 2017).

This review will address two aims: (1) a qualitative synthesis of empirical literature examining the structure, content and context of online far‐right propaganda, and (2) a quantitative synthesis of empirical literature examining the impact of online far‐right propaganda on audiences. By consolidating the available literature on the content, structure, context, and impact of far‐right propaganda, this review will provide an overview of the available evidence on the composition of far‐right propaganda and its impact on audiences. This analysis will include experimental research, qualitative research, and reports that detail empirical findings relevant to this review.

### 1.2 Defining Content, Structure, Context and Impact

This review aims to synthesise the literature on the content, structure, context, and impact of far‐right propaganda. It is hoped that this review will shed light on commonalities across far‐right propaganda campaigns and ultimately aid in the formation of effective counter‐narrative interventions specific to far‐right propaganda. Our operational definitions of content, structure, context and impact are detailed below, guided by the work of Jowett and O'Donnell (2012).

When referring to *content and structure* here, we are interested in the core components of the propagandistic messaging – the ‘what’ and ‘how’ of the propaganda effort. Specifically, what ideological themes, narratives and persuasive techniques are employed by authors of far‐right propaganda? For example, Zhang and Davis (2022) analyse themes and narratives specific to reactionary conservative far‐right propaganda from the United Kingdom and Australian publications. The authors identify specific narratives employed by propagandists (e.g., the ‘Great Replacement Theory’, anti‐multicultural, and anti‐immigration narratives) in their communication of the ideological themes of birth‐cultural nationalism and social dominance orientation (Zhang and Davis 2022). When synthesising data related to the *content* of far‐right propaganda, we will be compiling the ideological themes, narratives, and propagandistic techniques identified by scholars in the construction of online far‐right messaging. In synthesising data relating to structure, we will address how these themes, narratives and techniques scaffold together within the message. For example, Sarma (2007) analysed the content and structure of Provisional IRA propaganda, noting that messages justifying murders typically centred around a dehumanising central allegation (e.g., that the victim was a drug‐dealer) and with other techniques serving to enhance the emotive content of the message.

When referring to *context*, we are referring to peripheral contextual components of far‐right propaganda – the ‘who’, ‘where’ and ‘when’ of the propagandistic effort. Guided by the analytical steps outlined by Jowett and O'Donnell (2012), we will isolate the social and historical context that forms the backdrop to the propaganda, as well as information about the propagandist and movement/organisation. Zhang and Davis (2022), for example, analyse propaganda specific to the period following the Black Lives Matter (BLM) protests in the United States, in which far‐right propagandists used text‐based articles and memetic images to encourage anti‐BLM and anti‐immigrant sentiment among the general public.

When referring to *impact*, we are concerned with the effect propaganda has on audiences. Specifically, what is the impact of exposure to far‐right propaganda on audiences' emotions, attitudes and behaviour? Here, we anticipate synthesising relevant studies such as Braddock et al.'s (2022), which indicated that exposure to online far‐right propaganda is associated with gratification when engaging in subversive online activity, including trolling and doxing.

Below, we define content, structure, context and impact in more detail, setting out how we will approach each in our study of far‐right online propaganda. For the purposes of this review, we draw on Jowett and O'Donnell's (2012) framework, which sets out a process for isolating key components of propagandistic messaging. We outline below how we have adapted and re‐organised each stage of this framework to the study of the content, structure, context and impact of online far‐right propaganda:

1. The ideology and purpose of the propaganda campaign.
2. The context in which the propaganda occurs.
3. Identification of propagandist.
4. The structure of the propaganda organisation.
5. The target audience.
6. Media utilisation techniques.
7. Special various techniques.
8. Audience reaction to various techniques.
9. Counterpropaganda, if present, and.
10. Effects and evaluation.

#### 1.2.1 Content and Structure – What Is Being Said, and How?

##### 1.2.1.1 The Ideology and Purpose of the Propaganda Campaign

Jowett and O'Donnell (2012) define ideology as a ‘set of beliefs, values, attitudes and behaviours’ (p. 35), which constitute the normative way of thinking within a group. In the context of this review, it can be assumed that all RWE propaganda will contain far‐right ideology. As part of our synthesis, we will extract summary data with regard to the specific ‘ideological subcurrents’ of far‐right ideology identified by authors in the messaging (e.g., nativist, Neo‐Nazi, etc.). To guide this categorisation of far‐right groups, we will use the far‐right ideological subcategories identified in the PIRUS data set (National Consortium for the Study of Terrorism and Responses to Terrorism [START] 2023). The PIRUS data set identifies 12 subcategories of far‐right ideology based on a quantitative data set of individuals in the United States who have been radicalised to the point of ideologically motivated criminal activity.

The purpose of the propaganda campaign refers to the desired behavioural or attitudinal change the propagandist seeks to inspire within the target audience. As such, our synthesis will note (where possible) the beliefs or behaviours being encouraged by the propagandist being studied in the primary research (e.g., making a financial contribution or establishing the legitimacy/authority of a group/leader).

##### 1.2.1.2 Media Utilisation Techniques

Jowett and O'Donnell (2012) emphasise the importance of understanding how propagandists use different types of media to communicate. Here, they reference a wide variety of media such as radio, television, billboards, speeches and flags, emphasising that analysis of media utilisation should examine ‘every possibility’ of media usage. However, given the focus of the current review, our conceptualisation of media utilisation will be narrower in scope than that of Jowett and O'Donnell (2012). Here, we are interested in media techniques specific to the online environment, such as texts, videos, and images. Our synthesis will include the extraction of summary data on the types of media utilisation identified by authors in the primary studies included in this review (e.g., video, article, meme, etc.).

##### 1.2.1.3 Special Various Techniques

This stage in the framework involves identifying specific persuasive techniques utilised in the construction of propaganda. As such, our synthesis will include a summary of techniques identified by the authors of primary studies included in this review (e.g., emotional appeals, use of symbolism/iconography). The definition of ‘special various techniques’ provided in the framework is vague, and Jowett and O'Donnell (2012) emphasise that propaganda is too complex to be limited to a list of techniques. Our analysis will attempt to hone this understanding of the persuasive characteristics of propaganda, with specific reference to studies of online far‐right propaganda, including specific narratives used to communicate this ideology (e.g., ‘Great Replacement Theory’, scientific racism, etc.; Pauwels 2021). Included studies will use various methods of analysis, and the resultant synthesis will reflect the most commonly used methodologies in the study of far‐right propaganda, as well as a summary of the techniques most frequently identified in studies of far‐right propaganda.

#### 1.2.2 Context – By Who, When, and Where?

This phase of the analysis seeks to inform our understanding of context variables of far‐right propaganda disseminated online. These variables detail who is composing the propaganda, where and when it is being disseminated, and who has been exposed to it.

##### 1.2.2.1 The Context in Which the Propaganda Occurs

The framework stresses the importance of understanding the historical and social climate in which propaganda is disseminated. As such, during this stage of analysis, we will extract key variables related to the country of origin, year of dissemination, and a brief description of significant historical/social events interpreted by the propagandist.

Given the focus of the study, all propaganda being analysed will have been disseminated online. Therefore, we will synthesise key variables relevant to the online context, including where the propaganda was hosted (i.e., site or app name) and what type of site or app this hosting service provides (e.g., encrypted chat, social media, etc.). A synthesis of this data should provide a summary of the far‐right spaces being studied most by researchers.

##### 1.2.2.2 Identification of Propagandist

This stage of analysis involves identifying the group or individual who has produced the messaging. Our synthesis will note (where possible) the names of political parties, groups, or public figures responsible for the messaging being analysed within the primary studies included in the review. The purpose of this stage is to categorise the messaging according to the group or (public) individual of origin. Given the decentralised nature of RWE online spaces, this may not always be possible.

Jowett and O'Donnell suggest attempting to identify anonymous sources of propaganda as part of this stage. This is not feasible in the context of this review. We will simply identify the propagandist as they have been identified by the primary research author(s).

##### 1.2.2.3 The Target Audience

Jowett and O'Donnell (2012) stress the importance of identifying variants of ‘audience selection’ used by the propagandist, and indeed, it cannot be assumed that the general public is the intended audience for all propaganda disseminated in the online space. During synthesis, we will extract any available summary data on the target audience for the included RWE propaganda. During synthesis, we will also note (where possible) whether or not propaganda was privately or publicly available. This distinction is important, as propaganda intended for recruitment (and publicly available to outgroup members) may differ from messaging intended for ingroup members (Hardy 2023; Williams and Tzani 2022). Furthermore, should the propaganda make calls to action targeted towards a specific group or demographic (e.g., young white men), we will include this information in our synthesis.

#### 1.2.3 Impact

##### 1.2.3.1 Audience Reaction to Various Techniques

In this stage of the framework, Jowett and O'Donnell emphasise the importance of analysing audience response to propaganda. In the context of this review, audience response is a measure of the impact or effect of the messaging on audiences. As such, we have chosen to subsume this stage under the ‘Effects and Evaluation’ stage of the framework, where we will extract summary data on the cognitive and behavioural impacts of propaganda analysed in the primary studies.

##### 1.2.3.2 Effects and Evaluation

This stage of propaganda analysis involves determining whether the messaging achieved its aims, or in other words, was successful in inducing behavioural, attitudinal or emotional change. This element of Jowett and O'Donnell's framework will be addressed by our synthesis of outcomes relevant to the impact of online far‐right propaganda on audiences. Here, we will extract and synthesise summary data on the cognitive and/or behavioural impact of exposure to propaganda on audiences. This objective will inform our understanding of the effects of exposure to online far‐right propaganda on audiences. We will categorise this impact as being related to (a) radicalisation of opinion, (b) radicalisation of action, or both.

We will exclude two of Jowett and O'Donnell's steps from our analysis: ‘An analysis of counter‐propaganda’ and ‘Exploring the structure of the organisation behind the propaganda’. The remaining 8 steps will be used; however, we present the codebook that will be used to extract the data in Appendix S1, and an example of this framework being used in analysing one study in Appendix S2.

### 1.3 How Exposure to Online Far‐Right Propaganda May Be Linked to These Outcomes

With the proliferation of online far‐right content, as well as the mainstreaming of far‐right politics, social scientists seek to develop an understanding of how consumption of online far‐right media affects offline outcomes, such as shifts in attitudes and behaviours. Previous studies in the broader counter‐terrorism literature investigate the link between the use of the internet and offline violence. There is great debate about the role of online communications as part of the radicalisation process, and it has been evidenced that offline ‘push’ and ‘pull’ factors also play a role in the radicalisation process (Mølmen and Ravndal 2021).

These ‘push’ and ‘pull’ factors influence whether or not an individual engages with extremist content online, and subsequently becomes persuaded by it (i.e., radicalised; Borum 2011; Neo 2019). ‘Push’ factors include pre‐existing vulnerabilities that motivate individuals to become involved in violent extremism (e.g., adverse socio‐political circumstances, poverty, injustice and state‐repression; Mølmen and Ravndal 2021; Vergani et al. 2020). ‘Pull’ factors include the characteristics of an extremist group that individuals may find attractive, such as the group's ideology, or opportunities for social belonging (Borum 2011; Vergani et al. 2020). Scholars such as Vergani and colleagues (2020) emphasise the importance of ‘personal factors’, including psychological characteristics and past traumatic experiences, which also create vulnerability to radicalisation. Models of online radicalisation, such as that of Neo (2019), argue that ‘push’ and ‘pull’ factors, along with individual psychological characteristics, determine the extent to which an individual engages with and is persuaded by extremist content online. According to these models, radicalisation to violent extremism cannot occur solely as a result of consumption of online extremist material – offline ‘push’ and ‘pull’ factors, as well as individual characteristics, also play a role in the process of radicalisation (Mølmen and Ravndal 2021).

However, evidence from the study of online radicalisation has shown that the internet plays an important role in the move towards violent action. An analysis of a US sample of violent extremists found that social media use among perpetrators is increasing, with 85% of violent extremists between 2015 and 2016 using the internet as part of their radicalisation trajectory (Jensen et al. 2018). As the field of far‐right studies continues to grow, evidence is mounting that this process is occurring to those exposed to far‐right propaganda disseminated online. Interviews with former right‐wing extremists demonstrate the link between online and offline behaviours, with participants highlighting the importance of the internet for recruitment and coordination of action (Gaudette et al. 2020). Another important link between far‐right online and offline behaviours is the extent to which far‐right actors monetise the propaganda they disseminate to raise funds (Squire 2021). This review will synthesise the available data which investigates this link between exposure to online far‐right propaganda and the radicalisation of opinion and action.

### 1.4 Why It Is Important to Do This Review

Research suggests that exposure and susceptibility to far‐right extremist material can affect later behaviour, including engagement in subversive online activity (Braddock et al. 2022). As the proliferation of far‐right propaganda continues across online spaces (Baele et al. 2020), social scientists have begun to explore ways to mitigate the harmful effects of this messaging. Specific interventions, such as counternarratives (Carthy et al. 2020), seek to inoculate audiences against narratives employed by extremist propaganda to mitigate behavioural or attitudinal impact on audiences.

At present, there are no systematic reviews specific to the content, structure, context or impact of far‐right extremist propaganda disseminated online. A previous review by Wolfowicz et al. (2022) assessed the impact of different types of media on the process of radicalisation, including online and offline sources from a variety of ideologies. Furthermore, a review in progress by Hassan et al. (2022) aims to assess the impact of exposure to hate speech on audiences and will include an analysis of hate content disseminated on and offline. The present review will deviate from these publications due to the specificity of its focus, solely on content disseminated online by far‐right groups and individuals. It is worth noting that far‐right propaganda may not always include hate speech, and the proposed review aims to synthesise studies of far‐right propaganda, regardless of whether the target propaganda contains hate speech.

Understanding the structure, context, content and impact of right‐wing extremist propaganda can inform the design and implementation of policies and practices that seek to prevent radicalisation into violent extremism. This can include the design of messages that seek to inoculate target audiences to the persuasive content of the propaganda (i.e., through counternarratives), and through community initiatives that seek to build resilience and critical thinking among target audiences (Carthy et al. 2020). The proposed review aligns with Goal 16 of the UN Sustainable Development Plan (SDP). Far‐right propaganda has a role in the incitement of violence and hatred against several vulnerable groups, including refugees, immigrants, and LGBTQ+ people. In line with Goal 16.1 of the SDP, this review aims to support efforts to reduce far‐right‐related violence by enhancing our understanding of far‐right propaganda, which can, in turn, inform counter‐narrative efforts.

Despite the best efforts of media regulators, evidence suggests that policies which enforce the censorship of online extremist content do not eliminate the presence of this material. A study by Williams et al. (2023) found that social media users reported exposure to extremist content during approximately 48.44% of their daily time spent using these platforms. In the absence of effective content moderation, practitioners and policymakers need to understand the impact that online extremist material is having on audiences. This review aims to achieve this objective in the highly relevant context of far‐right extremist propaganda.

Online far‐right propaganda is particularly accessible to young people, for whom the online world is an inherent part of socialising (Centre for the Study of Democracy 2021). Research has identified the role of specific narratives used to target young audiences, which aim to influence aspects of an individual's identity (e.g., values and beliefs) (Kruglova and White 2025). For practitioners working with radicalised youth (in forensic settings) or young people at risk of exposure (in educational settings), a synthesis exploring the narratives and persuasive techniques used in online far‐right messaging would be valuable in working with individuals exposed to and potentially adopting these ideologies. It is important for practitioners to understand the narratives underlying these ideologies to effectively interrogate those beliefs with their clients.

Evidence of the efficacy of counternarrative interventions targeting extremism is mixed (Carthy et al. 2020; Treacy et al. 2024). A review of counternarratives by Carthy et al. (2020) found ‘discouraging evidence’ that these interventions can successfully target primary outcomes related to violent extremism, such as intentions to act violently. Furthermore, in their review of ‘live’ counternarrative campaigns targeting violent extremism, Treacy et al. (2024) noted that a majority of campaigns did not report the use of any guidelines or theory in the design or implementation of the intervention.

These findings emphasise the need for P/CVE practitioners, policymakers, and researchers to explore alternative methods of countering violent extremist messaging that are informed by theory and empirical research to target outcomes related to violent extremism effectively. To generate effective interventions that reduce the impact of far‐right propaganda, practitioners and policymakers involved in intervention design and implementation must have a nuanced understanding of the structure, content and context of far‐right propaganda campaigns across sub‐ideologies. This review aims to provide a synthesis of empirical evidence regarding online far‐right messaging to create a detailed understanding of the composition of these messages, which will aid the development of novel and effective interventions.

## 2 Objectives

This review will address two aims:

1. A qualitative synthesis of literature on the composition of online far‐right propaganda, guided by the following specific objectives:
  i. What is the content (i.e., themes) of online far‐right propaganda, and how does this differ across ideological subgroups?
  ii. What is the structure of online far‐right propaganda, and how does this differ across ideological subgroups?
  iii. What is the context of these messages (i.e., where, when and by whom were they posted?)
2. A quantitative synthesis of literature examining the impact of exposure to online far‐right propaganda, guided by the specific objective:
  i. What impact does exposure to online far‐right propaganda have on audiences, with reference to the radicalisation of opinion and/or action?

## 3 Methods

### 3.1 Criteria for Considering Studies for This Review: Objectives 1, 2 and 3 (Qualitative Synthesis)

Studies relevant to Objectives 1, 2 and 3 will include empirical qualitative studies that systematically analyse the content, structure and context of online far‐right propaganda.

### 3.2 Criteria for Considering Studies for This Review: Objective 4 (Quantitative Synthesis)

Studies relevant to Objective 4 will include experimental and quasi‐experimental studies assessing the cognitive and/or behavioural impact of exposure to online far‐right propaganda.

### 3.3 Types of Participants: Objectives 1, 2 and 3 (Qualitative Synthesis)

Authors of propaganda who can be described as ‘far‐right’ according to the umbrella concept defined by Pirro (2022) and the European Centre for Populism Studies, that is: Groups must be categorised as illiberal‐democratic (‘radical right’) or anti‐democratic (‘extreme right’). Screeners will allow for alternative labels (e.g., extreme right) if groups fall within the far‐right umbrella (as agreed upon by two reviewers).

### 3.4 Types of Participants: Objective 4 (Quantitative Synthesis)

Participants of experiments relevant to Objective 4 will include people who have been exposed to far‐right propaganda disseminated online (or simulated in an online experiment or quasi‐experiment), and where the impact of the exposure has been assessed within the studies. Control groups in such experiments will include participants not exposed to far‐right propaganda, and instead exposed either to neutral messaging or another form of extremist messaging. We envision categorising these groups based on control type (e.g., no messaging, neutral messaging, or non‐far‐right extremist messaging).

### 3.5 Types of Outcomes: Objectives 1, 2 and 3 (Qualitative Synthesis)

Outcomes relevant to Objective 1 (Content), Objective 2 (Structure) and Objective 3 (Context) will be the results of the qualitative analysis undertaken by authors of the primary research. This analysis must be replicable (as agreed upon by two coders). The data will be extracted with guidance from the framework detailed in Section 1.2.

### 3.6 Types of Outcomes: Objective 4: (Quantitative Synthesis)

Outcomes related to the impact of far‐right propaganda will include measures of the effect of exposure on radicalisation of opinion and radicalisation of action. Radicalisation of opinion will encompass impacts on attitudinal and emotional outcomes; that is, impacts on cognitive outcomes associated with exposure to far‐right online propaganda. For example, exposure to right‐wing anti‐immigration content has been demonstrated to influence implicit and explicit attitudes towards migrants in certain subpopulations (Matthes and Schmuck 2015). Outcomes related to radicalisation of action will include behavioural impacts on audiences exposed to far‐right propaganda, for example, violence (Pauwels and Schils 2014) and subversive online activity (Braddock et al. 2022). Table 1 provides a list of outcomes from each outcome domain (attitudinal, emotional, and behavioural) that have been identified in the literature, along with operational definitions and examples of how each outcome has been measured.

**Table 1:** Outcomes relevant to Objective 4.

Outcome domain	Outcome	Definition	Example of measurement
Attitudinal	Attraction towards extremist groups	Self‐reported attraction to the extremist group.	In Frischlich et al. (2019), measured via eight items on a 7‐point Likert scale.
Attitudinal	Implicit attitudes	Unconscious and automatic biases that participants hold about outgroups (e.g., immigrants).	In Matthes and Schmuck (2015), measured using an online version of the Implicit Association Test (IAT).
Attitudinal	Opinion extremism/agreement with extremist statements	Self‐reported agreement with radical views.	In Wojcieszak (2010), respondents rate statements on a scale from 1 to 7 (7 being most extreme; e.g., ‘All non‐white people who are now in the US should be deported and not allowed back into the country’). In Frischlich et al. (2019), measured via agreement with eight items on a 7‐point scale.
Attitudinal	Prejudice/explicit attitudes	Self‐reported prejudice towards outgroup members (e.g., anti‐Semitism, racism).	In Lee and Leets (2002), measured via response to three items on Likert Scale. In Matthes and Schmuck (2015) measured via three items on a 7‐point scale.
Attitudinal	Perceptions of one‐sidedness	Perceived one‐sidedness of extremist propaganda.	In Rieger et al. (2019), measured by response to items via 4‐point scale.
Attitudinal	Perceptions of source credibility	Perceived credibility of the source/authors of the propaganda.	In Braddock et al. (2022) measured via response to six 7‐point semantic differentials.
Attitudinal	Message acceptance	Self‐reported acceptance/agreement with messages present in propaganda.	In Lee and Leets (2002), via a semantic differential scale. In Rieger et al. (2013), five items on a 4‐point scale.
Attitudinal	Perceptions of persuasiveness	Perceived persuasiveness of extremist propaganda.	In Rieger et al. (2019), measured by response to items via 4‐point scale.
Emotional	Gratification	Self‐reported feelings of gratification (satisfaction, reassurance) in response to extremist propaganda.	In Braddock et al. (2022), measured via two items on a 7‐point Likert‐type scale.
Emotional	Intergroup anxiety	Self‐reported anxiety when interacting with individuals from different social groups.	In Matthes and Schmuck (2015), using a modified version of the Intergroup Anxiety Scale (IAS).
Emotional	Aversion	Self‐reported feelings of dislike/aversion in response to extremist propaganda.	In Rieger et al. (2019), measured via Modified Differential Affect Scale (M‐DAS) scale items.
Emotional	Anger	Self‐reported feelings of anger/irritation/frustration in response to propaganda.	In Braddock et al. (2022), measured via three items on a 7‐point Likert‐type scale.
Emotional	Guilt/shame	Self‐reported feelings of guilt/shame in response to extremist propaganda.	In Rieger et al. (2019), measured via Modified Differential Affect Scale (M‐DAS) scale items.
Emotional	Interest	Self‐reported interest in response to extremist propaganda.	In Rieger et al. (2019), measured via Modified Differential Affect Scale (M‐DAS) scale items.
Behavioural	Intentions to amplify/further engage with extremist content.	Self‐reported intentions to amplify, further engage with, and/or disseminate extremist propaganda.	In Frischlich et al. (2019), two items on Likert scale (i.e., ‘I would post the video’, ‘I would watch more videos like this’).
Behavioural	Political violence towards property	Self‐reported engagement in violence towards property (damaging or destroying property) for political reasons.	In Pauwels and Schils (2014), measured via the scale for nonconventional/illegal political participation. Respondents asked to respond to six items (e.g., if they had ever ‘written on a wall a political message or politically oriented graffiti’).
Behavioural	Political violence towards persons	Self‐reported engagement in violence towards other people for political reasons.	In Pauwels and Schils (2014), participants asked to respond to items on a Likert scale. (e.g., ‘threatened someone on the streets because of political or religious belief’ or ‘hit a foreigner’).
Behavioural	Subversive online activities	Self‐reported engagement in subversive online activities (e.g., trolling, doxxing, use of alt‐tech, and use of encrypted communication).	In Braddock et al. (2022), measured via response to items on 4‐point scale (1 = never, 4 = often).
Behavioural	Support intention	Self‐reported intentions to support an extremist group (e.g., financial contributions, store weapons).	In Braddock et al. (2022), four items measured via 7‐point scale.

### 3.7 Types of Studies: Objectives 1, 2 and 3 (Qualitative Synthesis)

Studies relevant to Objectives 1, 2 and 3 will include qualitative studies involving thematic analysis, narrative analysis or another approach to the systematic analysis of text (with this being met where the approach to analysis is sufficiently detailed to enable replication as agreed by two reviewers). Studies must report on first‐hand empirical findings, and publications which do not fulfil these criteria (e.g., editorial) will be excluded from review.

### 3.8 Types of Studies: Objective 4 (Quantitative Synthesis)

The types of studies relevant to Objective 4 will include quantitative experimental studies which investigate the impact of exposure to online far‐right propaganda on audiences. We will synthesise summary data from both experiments and quasi‐experiments relevant to this objective. We are guided by previous Campbell Collaboration reviews in identifying study designs which will be eligible for inclusion (e.g., Madriaza et al. 2025), namely:

– Experimental designs: We will include between‐subject experiments (or randomised control trials; RCTs), in which at least one experimental group was exposed to online far‐right propaganda. True experimental designs will assess the impact of exposure to online far‐right propaganda compared with a control group (i.e., participants not exposed to far‐right propaganda), by randomly allocating participants into treatment (exposure to far‐right propaganda) and control groups.
– Quasi‐experimental designs: We will include quasi‐experiments with two or more groups, in which at least one group is exposed to online far‐right propaganda, and compared with control groups (or groups exposed to another type of messaging). Eligible quasi‐experiments must compare at least one outcome between participant groups. These studies will include studies comparing a group before and after exposure to online far‐right propaganda, retrospective case‐control studies, and comparative studies employing a post‐test‐only design. We anticipate that quasi‐experiments relevant to this objective will compare exposure to online far‐right propaganda with exposure to another type of extremist material, differing either by context (e.g., offline far‐right propaganda) or content (e.g., Jihadi propaganda). For example, Rieger et al. (2019) compare the impact of right‐wing extremist and Islamist extremist videos on cognitive outcomes in audiences. Quasi‐experiments may also compare exposure to online far‐right propaganda to exposure to neutral, non‐propagandistic messaging. Quasi‐experiments relevant to this objective may also utilise non‐random assignment based on participants' prior exposure to online far‐right propaganda. For example, participants may be asked to self‐report prior exposure to online far‐right propaganda and their responses will be compared with those of participants who report no prior exposure to online far‐right propaganda.
– Cross‐sectional and longitudinal correlational studies: Studies with these designs will include studies based on a single sample, where an association is established between variations in exposure to online far‐right propaganda and at least one outcome of interest.

We anticipate that some experimental designs may feature ‘simulations’ of online far‐right propaganda (e.g., participants may be exposed to simulated/synthetic forms of far‐right propaganda). Simulations which do not include real‐world subjects or data sets (e.g., mathematical or computational models) will not be eligible for inclusion. However, we will include studies which expose participants to simulations or manipulations of existing far‐right propaganda. For example, some studies may use propaganda which has been generated by the research team or may modify existing propaganda for experimental purposes.

### 3.9 Inclusion and Exclusion Criteria

One search will be carried out to capture literature relevant to all objectives, as we anticipate some studies will be relevant to more than one objective. During the screening stage, we will ask reviewers to indicate whether results are relevant to the qualitative or quantitative synthesis. At this stage, the following exclusion and inclusion criteria will apply (Table 2).

**Table 2:** Inclusion and exclusion criteria.

	Inclusion	Exclusion
Document type	Empirical research (e.g., journal article, report) will be included for review.	Studies which do not report on empirical findings (e.g., newspaper articles) will be excluded from review.
Year of publication	Reports have identified that far‐right groups have been online since the early 1990s (Conway et al. 2019). As such, we will limit our results to 1990 onwards.	Studies published before 1990 will be excluded from review.
Exposure	Propaganda included in a study must come from a group or groups which can be defined as part of the ‘far‐right’ umbrella (as per the definition put forward by the European Centre for Populism Studies).	Studies which do not analyse far‐right groups/propaganda will be excluded from review. NOTE: Not all papers will use the term ‘far‐right’ – allow for alternative labels (e.g., extreme‐right, ultra‐right, etc.).
Context	Propaganda included in a study must have been disseminated online.	Propaganda disseminated solely offline (e.g., physical newsletter, poster campaigns) will be excluded from review. In the case of propaganda which has been disseminated both on‐ and off‐line, the propaganda must have been disseminated more than 50% online in order for the study to be eligible for inclusion.
Outcomes relevant to the qualitative synthesis (Objectives 1, 2 and 3)	Studies must include an analysis of the content, structure, and/or context of the target propaganda. Studies must include an analysis of the propaganda itself to be included in the review (e.g., narrative, thematic, and other forms of content analysis).	Studies which focus solely on non‐content/structure or ‐context properties of propaganda (e.g., network analysis which analyse platforms/URLS used by groups) will be excluded from review.
Outcomes relevant to the quantitative synthesis (Objective 4)	Studies which measure behavioural, emotional and/or attitudinal outcomes following exposure to online far‐right propaganda will be included in the review. No limits will be placed on which behavioural, emotional, and/or attitudinal outcomes will be included. It is anticipated that cognitive outcomes will include attitudinal and/or emotional impact (e.g., measures of fear, anger, outgroup sentiment, implicit biases, etc.). It is anticipated that behavioural outcomes will include primary and secondary outcomes related to violent extremism (e.g., violence, recruitment, fundraising, providing financial support, subversive online activity, voting behaviours, etc.). See Table 1 for a list of outcomes relevant to Objective 4.	No limits will be placed on which behavioural, emotional, and/or attitudinal outcomes will be included for review.
Design relevant to the qualitative synthesis: Objective 1 (Content), Objective 2 (Structure) and Objective 3 (Context)	Studies which analyse the content and context of far‐right propaganda will be included in the review. Studies must provide details on the approach deployed (e.g., content analysis, thematic analysis, etc.) or otherwise set out a transparent and replicable approach to the analysis as may arise where a bespoke approach is adopted.	
Design relevant to the quantitative synthesis (Objective 4, Impact)	Studies which investigate the relationship between exposure to online far‐right propaganda and behaviour, emotional and/or attitudinal outcomes will be included in the review. These may include experimental or quasi‐experimental (e.g., cross‐sectional, longitudinal, etc.) research assessing the behavioural, emotional, and/or attitudinal impact of exposure to far‐right propaganda disseminated online.	

### 3.10 Search Methods for Identification of Studies

The search strategy for the proposed review combines recommendations from the Cochrane‐Campbell Handbook for Qualitative Evidence Synthesis (Chapters 5 and 6), Cochrane Online training, other resources specific to systematic review and meta‐analysis (MacDonald et al. 2024), and expertise from the review team from work on past reviews (Sarma et al. 2022; Carthy et al. 2020). A specialist librarian from the University of Galway was consulted regarding database and search term selection.

Search Strategy:

The search strategy for this review will be comprised of four activities:

1. Targeted searches of relevant databases using keywords (Tables 3 and 4).
2. Hand searches of publications and other outputs from relevant professional agencies and research groups (Table 5).
3. Backward and forward citation analysis.
4. Consulting with experts in the area of extremist propaganda.

**Table 3:** Databases.

Platform	Database	#
Scopus.com (Elsevier)	Scopus	1
ProQuest	Dissertations and Theses Global	2
	Applied Social Sciences Index and Abstracts (ASSIA)	3
	Worldwide Political Science Abstracts	4
EBSCOhost	PsycInfo	5
	SocINDEX with full‐text	6
	Criminal Justice Abstracts	7
	PsycEXTRA	8
Web of Science	Social Sciences Citation Index	9
	Conference Proceedings Citation Index‐ Social Science and Humanities	10
	Emerging Sources Citation Index	11

**Table 4:** Search terms.

Search domain	Concept	Keywords
Title, abstract, keywords	Target group (*far‐right*)	alt‐right OR ethno‐nationalis* OR ‘extreme right’ OR ‘extremist right wing’ OR ‘far right’ OR fascis* OR ‘hard‐right’ OR ‘neo‐nazi’ OR nativis* OR ‘neo‐fascis*’ OR ‘racial nationalis*’ OR ‘radical right’ OR ‘right wing extremis*’ OR ‘right‐wing populis*’ OR ‘trumpis*’ OR ‘ultra right’ OR ‘white nationalis*’ OR ‘white supremac*’
Title, abstract, keywords	Issue/phenomenon/exposure (*online propaganda*)	‘Alternative historical accounts’ OR branding OR disinformation OR ‘dis information’ OR ideolog* OR messaging OR ‘media communication*’ OR misinform* OR narrative* OR persua* OR ‘persuasive communication*’ OR ‘political advertis*’ OR ‘political communication*’ OR propagand* OR ‘psychological operation*’

#### 3.10.1 Electronic Searches

We (M. D. and K. C.) will execute a search of the literature via the electronic platforms and databases below. One search will be carried out to capture literature relevant to all objectives, as we anticipate some results will be relevant to more than one objective. Empirical reports have identified that far‐right groups have been online since the early 1990s (Conway et al. 2019). As such, we will limit our search results to 1990 onwards. The chosen databases for this search encompass a wide range of peer‐reviewed works from a variety of disciplines, as well as unpublished grey literature, including dissertations and theses. Both Scopus and Applied Social Sciences Index and Abstracts (ASSIA) include grey literature.

Given the specificity of the review objectives, and based on pilot searches conducted by the research team, it is anticipated that only a small number of studies will be eligible for review. As such, this strategy has been purposefully designed to be broad in scope to best capture relevant records for inclusion in the review. We anticipate this search will generate a large number of results, and we will screen these results with the help of AI active machine learning (AML).

We will employ a search syntax specifically tailored to each search engine using the Polyglot translation tool. These search syntaxes will be composed from the search terms below. These terms will be applied to ‘title, abstract and keywords’ in each case:

An example search of the database Scopus is as follows:

TITLE‐ABS‐KEY(alt‐right OR ethno‐nationalis* OR ‘extreme right’ OR ‘extremist right wing’ OR ‘far right’ OR fascis* OR ‘hard‐right’ OR ‘neo‐nazi’ OR nativis* OR ‘neo‐fascis*’ OR ‘racial nationalis*’ OR ‘radical right’ OR ‘right wing extremis*’ OR ‘right‐wing populis*’ OR ‘trumpis*’ OR ‘ultra right’ OR ‘white nationalis*’ OR ‘white supremac*’) AND TITLE‐ABS‐KEY (‘Alternative historical accounts’ OR branding OR disinformation OR ‘dis information’ OR ideolog* OR messaging OR ‘media communication*’ OR misinform* OR narrative* OR persua* OR ‘persuasive communication*’ OR ‘political advertis*’ OR ‘political communication*’ OR propagand* OR ‘psychological operation*’).

Titles and abstracts for records captured in this search will be exported to the reference management software *DistillerSR* to facilitate the screening process.

#### 3.10.2 Searching Other Resources

To provide coverage of government reports and other publications relevant to this review, the following websites will be hand‐searched:

**Table 5:** Resources for hand‐searching.

Agency/Group	Website/URL
Centre for Research and Evidence on Security Threats	https://crestresearch.ac.uk/
International Centre for Counterterrorism (ICCT)	https://www.icct.nl/publications
European Consortium for Political Research (ECPR)	https://ecpr.eu/
European Commission Radicalisation Awareness Network	https://home-affairs.ec.europa.eu/networks/radicalisation-awareness-network-ran_en
International Centre for the Study of Radicalisation (ICSR)	https://icsr.info/
Institute for Strategic Dialogue (ISD)	https://www.isdglobal.org/
VOX‐Pol Research Network	https://voxpol.eu/
European Expert Network on Terrorism Issues	https://www.european-enet.org/
Centre for Research on Extremism (C‐Rex)	https://www.start.umd.edu/
	https://www.sv.uio.no/c-rex/english/
International Centre for the Study of Radicalisation (ICSR)	https://icsr.info/
International Centre for the Study of Violent Extremism	https://icsve.org/
National Consortium for the Study of Terrorism and Responses to Terrorism	https://www.start.umd.edu/
The Addressing Violent Extremism and Radicalisation to Terrorism (AVERT) Research Network	https://www.avert.net.au/

We (M. D., K. C. and K. M. S.) will also review past systematic reviews in this area to identify papers relevant to our review. We will identify these and other primary research relevant to our review via backward and forward citation analysis. The bibliographies of eligible papers and systematic reviews will be examined for literature that may meet our inclusion criteria (backward citation analysis/harvesting). We will also identify papers which cite relevant references via forward citation analysis. For example, where a paper meets our inclusion criteria and is indexed on Scopus, the ‘Citing articles’ option will be used to identify any records in the database which cite the target article. We will supplement this further with forward‐citation analysis via Google Scholar. Records identified via backward or forward citation analysis will be labelled as ‘citation analysis’ in the accompanying PRISMA chart.

### 3.11 Data Collection and Analysis

#### 3.11.1 Overview

The screening process will be conducted by two reviewers (M. D. and K. C.) using DistillerSR. Should searches capture too large a number of results to allow for manual screening (> 10,000), the team will use the DistillerSR AI AML function to facilitate faster title/abstract screening. The DistillerSR AML is a validated AI screening tool, which, when trained on a proportion of results, can reduce the screening burden (Fabiano et al. 2024; Hamel et al. 2020). The AML screening feature will be trained based on initial inclusion and exclusion decisions made by two screeners (M. D. and K. C.). Following automated removal of duplicate entries, the two reviewers will begin the training phase of the screening process. The inclusion/exclusion criteria outlined in Section 3.9 will apply at this stage.

This training phase of the screening process will be guided by the work of Hamel et al. (2021). To train the algorithm, a set of records (2% of all records obtained in the search) will be shuffled and randomly selected. This will make up the training set. These records will be manually double‐screened by two reviewers (M. D., K. C.). Following this initial screening, interrater reliability (IRR) will be calculated, and disagreements will be discussed and resolved. Once a satisfactory level of agreement on this initial set is reached (kappa of 0.8), the training stage of screening will be considered complete. After this training phase, DistillerSR will score and rank the remaining records based on the likelihood of inclusion. The screening process will then continue with each additional record being screened by both reviewers. DistillerSR will re‐score and re‐rank papers following the completion of every 2% of records. To ensure optimal performance of the prioritisation tool, reviewers will strive to review records at the same pace and will adhere to daily goals of the number of records to be screened. Records will be double‐screened at each phase of the screening process. IRR will be calculated automatically by the DistillerSR software following each screening phase.

The DistillerSR AML includes a predictive reporting tool, which estimates the number of relevant records identified, and allows reviewers to set a ‘stop screening’ rule, which is typically set at 95% recall (Campos et al. 2024; Hamel et al. 2021). However, due to the broad scope of this review and the diffuse nature of the literature, we will move to single screening when 95% recall has been reached. This will allow the team to identify clusters of records further down the list of results that may have been missed if a stop screening rule were in place.

#### 3.11.2 Full‐Text Screening

Records that graduate from the initial title and abstract screening will be subject to a full‐text screening. These records will be assessed for eligibility based on the review objectives of (1) Content, (2) Structure, (3) Context and/or (4) Impact. Reviewers will exclude documents based on the criteria outlined in Section 3.9. Reviewers will also be asked to indicate which objective(s) the record is relevant to to expedite the data extraction process. Where uncertainty or disagreement arises, the authors will resolve final inclusion decisions via discussion, with a third author if necessary (K. M. S.).

IRR will be calculated via the use of the prevalence‐adjusted and bias‐adjusted kappa (Byrt et al. 1993) at the beginning, middle, and end of the screening process.

#### 3.11.3 Data Extraction and Management

Detailed extraction tables will be generated, which capture key information from each study. This data will include authors, date of publication, design, aim/purpose of the study, specific far‐right group included in the study, comparison group (if any), and results. See Appendix S1 for the codebook which will be used for data extraction. See Appendix S2 for examples of extraction tables for studies relevant to each objective.

#### 3.11.4 Machine Translation

Far‐right groups operate across Europe and the wider world. As such, some studies eligible for inclusion in this review may require machine translation in order for them to be synthesised by the English‐speaking review team. Abstracts and full‐texts captured by the search will be translated to facilitate the screening process. We will adopt the procedure of Balk et al. (2013), who use Google Translate for machine translation. We anticipate addressing limitations of this method, as well as limitations of machine translation of thematic/content analysis in general, as part of the discussion segment of the review.

#### 3.11.5 Dealing With Missing Data

If quantitative data needed to generate effect sizes for the meta‐analysis component of the review are missing from an included paper, the corresponding author will be contacted by a member of our team (M. D.). Where access to this quantitative data is unavailable, the relevant paper will be excluded from meta‐analysis but retained as part of the qualitative synthesis.

#### 3.11.6 Assessment of Risk of Bias in Included Studies

Risk of Bias assessments will be conducted using Joanna Briggs Institute Quality Assessment Checklists relevant to each study design. This will include assessment using tools tailored to qualitative (Lockwood et al. 2015), quasi‐experimental (Barker et al. 2024), and randomised control trial experimental designs (Barker et al. 2023). Items for each of these tools are presented in Appendix S2. Should studies of other experimental designs be included in the final analysis, we will supplement this list of tools with the appropriate JBI Checklists.

#### 3.11.7 Criteria for Determination of Independent Findings

We anticipate that some eligible studies will include secondary analysis of data from previous studies. Before data extraction, the team will assess if there are any overlaps in study samples. Research documents using the same sample will be treated as a single study, to ensure that only data from unique samples are used for meta‐analysis.

To address dependency, and to enable the synthesis of data using different measures within the same outcome domain (e.g., different measures of attitudinal change within the ‘cognitive radicalisation’ domain), we will use an aggregation approach (Carthy et al. 2020; Gucciardi et al. 2021). We describe this in further detail in Section 3.12.1.

### 3.12 Data Synthesis

To synthesise all qualitative data captured by our search, we will conduct a framework synthesis to integrate findings relevant to Objective 1 (content), Objective 2 (structure) and Objective 3 (context). The framework synthesis process will be guided by the work of Ritchie et al. (2013). This guidance outlines the following stages of data analysis using the framework method:

1. Familiarisation
  – This stage involves immersion in the data set and becoming familiar with the data. At this stage, we will begin to identify topics or themes of interest that are recurrent across the data.
2. Constructing an initial thematic framework
  – At this stage, we will apply the Jowett and O'Donnell framework as outlined in Section 1.2. We will populate this framework with studies we know to be eligible (i.e., ‘seed studies’).
3. Indexing and sorting
  – The framework will be used to annotate and label the data. Data will be indexed or coded with the themes and subthemes identified in the initial framework. Following indexing, data will be sorted so individual themes and subthemes can be viewed as a whole, to allow for detailed analysis of individual themes and subthemes.
4. Reviewing data extracts
  – The indexed and sorted data will be reviewed to see if the extracted data fits the initial thematic framework. The research team will then assess if labels need to be amended or reapplied to the data, and whether extracted data has been assigned to appropriate themes and subthemes.
5. Data summary and display
  – Summaries will be written for each theme, subtheme, and study included in the review. These summaries will then be displayed by theme and study in a set of matrices.
6. Abstraction and interpretation
  a. Following the labelling, review, and display of the extracted data, the abstraction and interpretation process will involve more analytical classification and categorisation of the data. Ritchie et al. (2013) suggest the following activities during this phase:
    i. Description: We will produce a qualitative account of each study's findings.
    ii. Developing categories: Themes and subthemes will be analysed at a deeper level, with relevant data extracts being categorised and mapped to reflect the range of views/experiences/breadth of the topic.
    iii. Mapping linkage: We will explore the ways in which different parts of the data interact or are connected with one another. For example, we will note whether associations exist between certain experiences described within the qualitative data and behaviours or cognitive outcomes.
    iv. Explanation: During this stage, we will develop an understanding of why and how certain elements of the data interact or are connected with one another. We will search for key processes or factors which account for any patterns of association observed in the data. These explanations will be based on explicit findings within the primary research, as well as logical inferences made by the research team about patterns within the data.

#### 3.12.1 Statistical Procedures for Meta‐Analysis

Studies relevant to Objective 4 (Impact) will be quantitative in design. If a minimum of two studies yield effect sizes of the same outcome construct, we will conduct a meta‐analysis of the results of each association between exposure to online far‐right propaganda and relevant outcomes. We anticipate that most studies will consider one independent variable (exposure/consumption of far‐right online propaganda) and one or more dependent variables (cognitive or behavioural outcomes). We will calculate effect sizes to indicate the strength of the relationship between these variables of interest. Summary statistics (i.e., correlation coefficients, data from regression models, etc.) will be extracted for this purpose.

For studies using continuous variables to compare experimental groups, or pre‐post scores, we will calculate standard mean differences (Cohen's *d*, Glass's delta or Hedges *g*) by extracting means, standard deviations and sample sizes from each study. For studies using dichotomous variables, we will report the logarithm of the odds ratio as the measure of effect size. For studies which provide correlation matrices showing bivariate correlations between variables of interest, we will transform Pearson's *r* to Fisher's *z* to calculate the effect size.

It is anticipated that some studies will report more than one effect size, such as in cases where multiple outcomes are measured within a single domain (e.g., attitudinal and emotional outcomes in the ‘cognitive radicalisation’ domain) or multiple methods to measure the same outcome are used (e.g., Implicit Association Test and self‐report measures for bias). In such cases where dependent effects are present in the literature, we will use an aggregation approach to address dependency (Carthy et al. 2020; Gucciardi et al. 2021). Where possible, we will report on comparable outcome measures. For example, attitudinal outcomes relating to misogyny can be measured using a variety of psychometric tools, including the Misogyny Scale (Rottweiler et al. 2024) and the Ambivalent Sexism Inventory (Glick and Fiske 1996).

Where sufficient data is available (minimum 10 eligible studies), subgroup analysis will be conducted to investigate the impact of methodological characteristics of studies on effect sizes. We anticipate these methodological characteristics to include the type of control group used (e.g., Jihadi propaganda or neutral messaging), study design (effects found in experimental vs. quasi‐experimental designs), and duration of follow‐up (for longitudinal studies, e.g., 2‐month interval in Weiss et al. 2024 and 2‐week interval in Lee and Leets 2002). We will also conduct subgroup analysis to investigate the impact of the type of propaganda used in the study on effect sizes. For example, we will compare the effect of different ‘ideological subcurrents’ of far‐right propaganda, if sufficient data is available. We will also conduct subgroup analysis based on the characteristics of participants in each study. For example, during age‐based subgroup analysis, we will categorise participants into discrete subgroups based on age (e.g., children [under 18], young adults [18–25]). This subgroup analysis will explore whether participants of specific subpopulations are more susceptible, or uniquely affected by, exposure to online far‐right propaganda. Previous research has identified generational patterns and a gender gap in support for far‐right groups (Harteveld et al. 2015; Rekker 2024). Subgroup analysis will enable us to see if these findings are reflected in the social science literature captured by our search.

Effect sizes will be analysed using the Comprehensive Meta‐Analysis software according to the random effects analytical model. A random effects model for meta‐analysis has been chosen as included studies will estimate different, yet related, effects of exposure within the domains of cognitive and behavioural radicalisation. Heterogeneity will be assessed using the L‐squared statistic, the tau‐squared statistic, and Cochrane's *Q* for random effects meta‐analysis.

#### 3.12.2 Integration of Qualitative and Quantitative Syntheses

**Figure 1:**
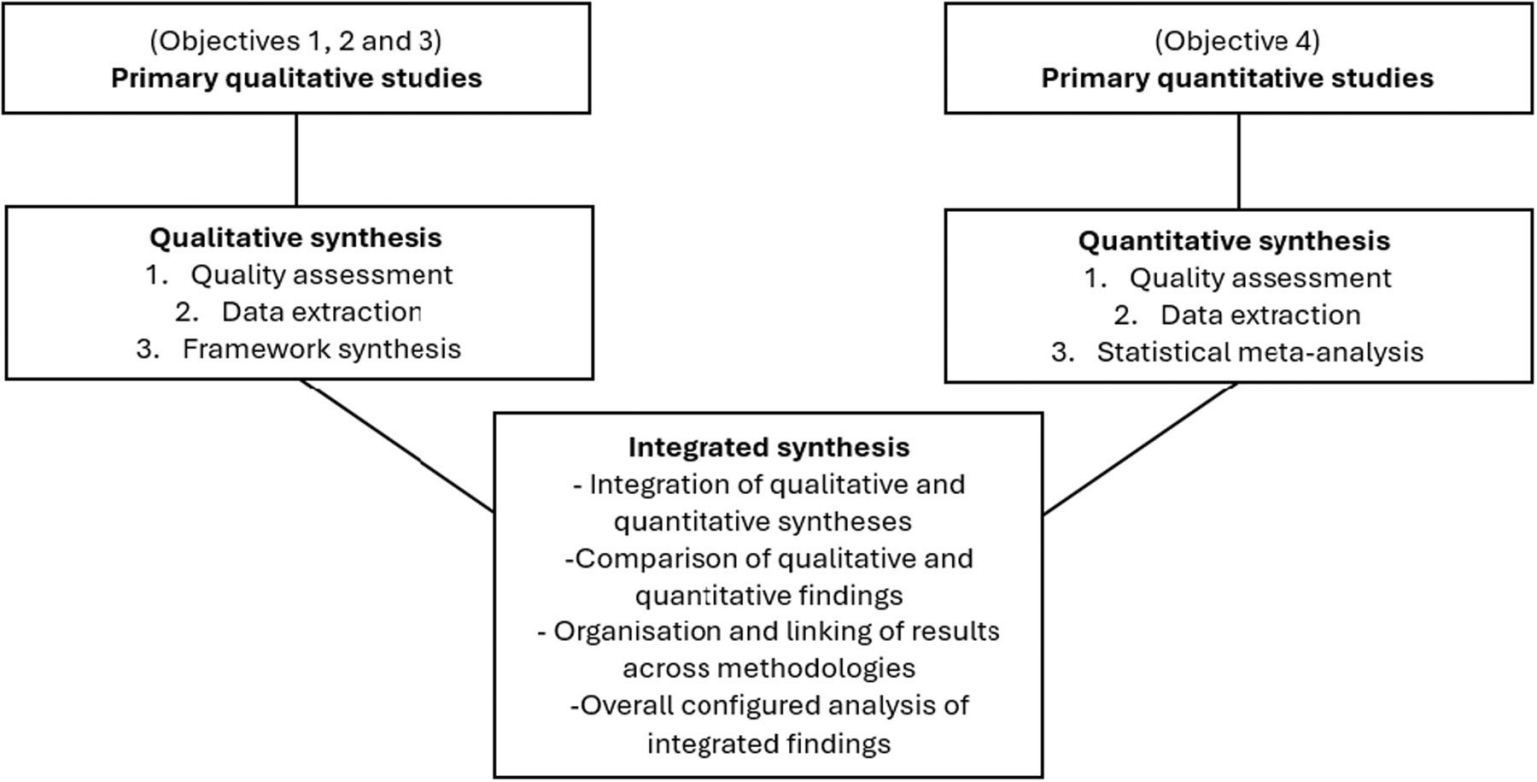
Integration of qualitative and quantitative syntheses.

Following the completion of the qualitative (framework synthesis) and quantitative (meta‐analysis) syntheses, these findings will be integrated into a final third synthesis. This final synthesis product will allow for the juxtaposition of qualitative and quantitative data relevant to the review objectives. Guided by the work of Stern et al. (2020) and Harden (2010), this configured synthesis product will enable us to see how results from qualitative studies may inform the interpretation of the quantitative data, or vice versa. See Figure 1 for an overview of the planned synthesis.

#### 3.12.3 Investigation of Heterogeneity

Heterogeneity will be assessed using the L‐squared statistic, the tau‐squared statistic, and Cochrane's *Q* for random effects meta‐analysis.

#### 3.12.4 Assessment of Publication Bias

Assessment of publication bias will be conducted via subgroup analysis of published and unpublished studies. Funnel graphs will be used to illustrate the findings of this analysis.

## Author Contributions

Each member of the team will make the following contributions. Content: Mia Doolan and Kiran M. Sarma. Systematic review methods: Mia Doolan and Katie Cox. Statistical analysis: Kiran M. Sarma and Mia Doolan. Information retrieval: Mia Doolan and Katie Cox.

## Conflicts of Interest

Dr. Kiran M. Sarma is an Associate Editor of the Campbell Collaboration and will be excluded from any editorial decisions concerning the proposed review. The other authors declare no conflicts of interest.

## Preliminary Timeframe

Review to be completed by September 2025.

## Plans for Updating This Review

This review will be updated by the primary author every 5 years to include data from newly published studies that meet the criteria for inclusion.

## Sources of Support

This review is part of a PhD project funded by Taighde Éireann – Research Ireland.

## Transparent Peer Review

The peer review history for this article is available at https://www.webofscience.com/api/gateway/wos/peer-review/10.1002/cl2.70076.

## Supporting information

Supp Materials.

## Data Availability

The authors have nothing to report.
