# Supplementary material for: Protocol: Understanding the Content, Context, and Impact of Far‐Right Extremist Propaganda Disseminated Online: A Systematic Review
Source: Campbell Syst Rev. 2025 Nov 14;21(4):e70076. doi: 10.1002/cl2.70076 (PMC12616883; doi:10.1002/cl2.70076)
Supplement: Supplementary file 1 — Supp Materials. [file CL2-21-e70076-s001.docx]

Appendix 1: Codebook for data extraction

| **Domain** | **Code** | **Code definition** | **Examples** |
| --- | --- | --- | --- |
| **Bibliographic Information** | Year | Year of publication |  |
|  | Author(s) | Authors of primary study |  |
|  | Aim/Purpose of study | Objectives of the primary study | Narrative analysis, content analysis  Experiment assessing impact of exposure to far-right propaganda |
| **Content and Structure** | Ideology | Specific “strain” of far-right ideology | Neo-Nazi, white supremacist, nativist. |
|  | Purpose | The desired cognitive or behavioural change encouraged by the messaging. | Providing financial support, recruitment, protest, etc.  NOTE: Purpose must be explicitly identified by authors in the primary study – do not infer purpose post-hoc. |
|  | Media Utilisation Techniques | Types of media used in the messaging. | Video, image, text, meme |
|  | Special Various Techniques | Persuasive techniques present in the messaging (as identified by qualitative analysis in the primary study). | Emotional appeals, expert witness, use of symbolism/iconography  Use of specific narratives (e.g. “scientific racism”, “great replacement theory”). |
| **Context** | Propagandist | Person or group responsible for the composition of the messaging | Public figure (Anders Breivik) or group name (English Defence League) |
|  | Context | 1. Country of origin, year of dissemination, brief description of significant historical/social events interpreted by the propagandist. 2. Site/App name, type of site/app used. | 1. England, 2019. Descriptions of mass immigration. 2. Twitter (Social Media). Telegram (encrypted chat). |
|  | Target Audience | Demographic being targeted by the propagandist as:   1. Explicitly stated in the messaging 2. Identified by authors of the primary study | Note where data was privately or publicly available (in-group or out-group communication).  Note explicit calls to action targeting specific groups (e.g, young white, European men). |
| **Impact** | Design | Experimental design used | Experimental, quasi-experimental, RCT (give specific descriptions) |
|  | Participants | Sample size, gender, age, etc. | N=404, 90% Male, 73% 18-35 y/o, 74% White |
|  | Propaganda | Brief description of propaganda used in the experiment | “Scientific racism”, male supremacy, antisemitic, anti-immigration, etc. |
|  | Outcome(s) | Outcomes measured within the experiment | Radicalisation of action: Violence, voting behaviours, recruitment, fundraising, providing material support, subversive online activity.  Radicalisation of opinion: Implicit and explicit attitudes, emotional outcomes, perception of source credibility, psychological reactance, implicit biases. |
|  | Results | Brief qualitative description of the results of the study | Exposure to scientific racism narratives significantly associated with implicit biases towards immigrants. |

Appendix 2: Extraction tables

Descriptive coding (Zhang & Davis, 2022)

Objectives 1 and 2: Content  and Structure

| Ideology | “Reactionary Conservative” |
| --- | --- |
| Ideological themes | Birth-cultural nationalism, post-fascist authoritarianism, Social dominance orientation (SDO) and Anti-politics populism |
| Narratives identified | “White genocide”, “Great Replacement Theory”, anti-white, anti-immigration, anti-multiculturalism, anti-Islam, anti-liberal left, anti-media, anti-BLM, pro-Christianity, freedom of speech, nationalism, law and order, white supremacy, white privilege. |
| Media utilisation | Text-based articles |
| Techniques identified | 1. “Justification, normalisation and legitimisation” of anti-multicultural sentiments. 2. Use of memetic communications (memes) as means of sarcasm or ironic humour. |

Objective 3: Context

| Author(s), Year | (Zhang & Davis, 2022) | |
| --- | --- | --- |
| Aim/Purpose of Study | Qualitative content analysis of 400 far-right online posts | |
| Context | Black Lives Matter protests in U.S. | |
| Propagandist/Group/Individual | 2Australian sources | 2 UK Sources |
|  | (1) The Unshackled  (2) XYZ | (1) British First  (2) Politicalite |
| Purpose | (1) Spread “anti-liberal left” and “white victimhood” narratives in the wake of BLM protests.  (2) Spread of “anti-Islam” narratives to promote anti-immigrant sentiment.  (3) Protest deplatforming by espousing virtues of freedom of speech. | |
| Target audience | General public – ingroup and outgroup | |

Descriptive coding (Objective 4: Impact)

| Author(s), Year | (Braddock et al., 2022) | |
| --- | --- | --- |
| Aim/Purpose of Study | Investigating the link between online misbehaviour and susceptibility to persuasion by far-right propaganda. | |
| Design | Experimental, one group post-test only | |
| Participants (n, gender, age, etc.) | Study 1 (N=404) | Study 2 (N=396) |
|  | 90% Male,  73% 18-35 y/o  74% White | 90% Male  78% 18-35 y/o  70% White |
| Propaganda used | Study 1 “Scientific Racism” | Study 2 “Male Supremacy” |
| Outcome(s) | Radicalisation of opinion: Gratification, Perception of Source Credibility, Psychological Reactance, Anger, Support Intention.  Radicalisation of action: Subversive online activity (doxxing, trolling, use of alt-tech, etc), Counter-arguing | |
| Results | Engagement in SOA predicts susceptibility to far-right propaganda of both “strains”. | |

Appendix 3 – Tools for assessing risk of bias

JBI Critical Appraisal Checklist for Qualitative Research

| RoB Assessor: | Date of Appraisal: | Record Number |
| --- | --- | --- |
| Study Author: | Study Title: | Study Year |

| Field | Response Options |
| --- | --- |
| Is there congruity between the stated philosophical perspective and the research methodology? | Yes  No  Unclear  Not Applicable |
| Is there congruity between the research methodology and the research question or objectives? | Yes  No  Unclear  Not Applicable |
| Is there congruity between the research methodology and the methods used to collect data? | Yes  No  Unclear  Not Applicable |
| Is there congruity between the research methodology and the representation and analysis of data? | Yes  No  Unclear  Not Applicable |
| Is there congruity between the research methodology and the interpretation of results? | Yes  No  Unclear  Not Applicable |
| Is there a statement locating the researcher culturally or theoretically? | Yes  No  Unclear  Not Applicable |
| Is the influence of the researcher on the research, and vice- versa, addressed? | Yes  No  Unclear  Not Applicable |
| Are participants, and their voices, adequately represented? | Yes  No  Unclear  Not Applicable |
| Is the research ethical according to current criteria or, for recent studies, and is there evidence of ethical approval by an appropriate body? | Yes  No  Unclear  Not Applicable |
| Do the conclusions drawn in the research report flow from the analysis, or interpretation, of the data? | Yes  No  Unclear  Not Applicable |
| **Overall Appraisal** |  |
| Comments |  |

JBI Checklist for Quasi -Experimental Studies

| RoB Assessor: | Date of Appraisal: | Record Number: |
| --- | --- | --- |
| Study Author: | Study Title: | Study Year: |

| Field | Comments/Justification | Categorical Appraisal |
| --- | --- | --- |
| **Internal Validity** | | |
| Bias related to temporal precedence  Is it clear in the study what is the “cause” and what is the “effect” (i.e. there is no confusion about which variable comes first)? |  | Yes  No  Unclear  Not Applicable |
| Bias related to selection and allocation    Was there a control group? |  | Yes  No  Unclear  Not Applicable |
| Bias related to confounding factors    Were participants included in any comparisons similar? |  | Yes  No  Unclear  Not Applicable |
| Bias related to administration of intervention/exposure    Were the participants included in any comparisons receiving similar treatment/care, other than the exposure or intervention of interest? |  | Yes  No  Unclear  Not Applicable |
| Bias related to assessment, detection and measurement of the outcome    Were there multiple measurements of the outcome, both pre and post the intervention/exposure?    Outcome 1:  Outcome 2:  Outcome 3:  Outcome 4:  Outcome 5:  Outcome 6:      Were the outcomes of participants included in any comparisons measured in the same way?    Outcome 1:  Outcome 2:  Outcome 3:  Outcome 4:  Outcome 5:  Outcome 6:      Were outcomes measured in a reliable way?    Outcome 1:  Outcome 2:  Outcome 3:  Outcome 4:  Outcome 5:  Outcome 6: |  | Yes, No, Unclear, N/A  Yes, No, Unclear, N/A  Yes, No, Unclear, N/A  Yes, No, Unclear, N/A  Yes, No, Unclear, N/A  Yes, No, Unclear, N/A                  Yes, No, Unclear, N/A  Yes, No, Unclear, N/A  Yes, No, Unclear, N/A  Yes, No, Unclear, N/A  Yes, No, Unclear, N/A  Yes, No, Unclear, N/A            Yes, No, Unclear, N/A  Yes, No, Unclear, N/A  Yes, No, Unclear, N/A  Yes, No, Unclear, N/A  Yes, No, Unclear, N/A  Yes, No, Unclear, N/A |
| Bias related to participant retention    Was follow-up complete and if not, were differences between groups in terms of their follow-up adequately described and analysed?      Outcome 1:  -Result 1:  -Result 2:  -Result 3:      Outcome 2:  -Result 1:  -Result 2:  -Result 3:      Outcome 3:  -Result 1:  -Result 2:  -Result 3:      Outcome 4:  -Result 1:  -Result 2:  -Result 3:      Outcome 5:  -Result 1:  -Result 2:  -Result 3:      Outcome 6:  -Result 1:  -Result 2:  -Result 3: |  | Yes, No, Unclear, N/A  Yes, No, Unclear, N/A  Yes, No, Unclear, N/A        Yes, No, Unclear, N/A  Yes, No, Unclear, N/A  Yes, No, Unclear, N/A        Yes, No, Unclear, N/A  Yes, No, Unclear, N/A  Yes, No, Unclear, N/A        Yes, No, Unclear, N/A  Yes, No, Unclear, N/A  Yes, No, Unclear, N/A        Yes, No, Unclear, N/A  Yes, No, Unclear, N/A  Yes, No, Unclear, N/A        Yes, No, Unclear, N/A  Yes, No, Unclear, N/A  Yes, No, Unclear, N/A |
| **Statistical Conclusion Validity** | | |
| Was appropriate statistical analysis used?    Outcome 1:  -Result 1:  -Result 2:  -Result 3:      Outcome 2:  -Result 1:  -Result 2:  -Result 3:      Outcome 3:  -Result 1:  -Result 2:  -Result 3:      Outcome 4:  -Result 1:  -Result 2:  -Result 3:      Outcome 5:  -Result 1:  -Result 2:  -Result 3:      Outcome 6:  -Result 1:  -Result 2:  -Result 3: |  | Yes, No, Unclear, N/A  Yes, No, Unclear, N/A  Yes, No, Unclear, N/A        Yes, No, Unclear, N/A  Yes, No, Unclear, N/A  Yes, No, Unclear, N/A        Yes, No, Unclear, N/A  Yes, No, Unclear, N/A  Yes, No, Unclear, N/A        Yes, No, Unclear, N/A  Yes, No, Unclear, N/A  Yes, No, Unclear, N/A        Yes, No, Unclear, N/A  Yes, No, Unclear, N/A  Yes, No, Unclear, N/A        Yes, No, Unclear, N/A  Yes, No, Unclear, N/A  Yes, No, Unclear, N/A |
| **Overall Appraisal** | **Comments** |  |

JBI Critical Appraisal Tool for Assessment of Risk of Bias for Randomized Controlled Trials

| RoB Assessor: | Date of Appraisal: | Record Number: |
| --- | --- | --- |
| Study Author: | Study Title: | Study Year: |

| Field | Comments/Justification | Categorical Appraisal |
| --- | --- | --- |
| **Internal Validity** | | |
| Bias related to selection and allocation    Was true randomization used for assignment of participants to treatment groups?      Was allocation to treatment groups concealed?      Were treatment groups similar at the baseline? |  | Yes  No  Unclear  Not Applicable      Yes  No  Unclear  Not Applicable    Yes  No  Unclear  Not Applicable |
| Bias related to administration of intervention/exposure    Were participants blind to treatment assignment?          Were those delivering the treatment blind to treatment assignment?        Were treatment groups treated identically other than the intervention of interest? |  | Yes  No  Unclear  Not Applicable      Yes  No  Unclear  Not Applicable        Yes  No  Unclear  Not Applicable |
| Bias related to assessment, detection and measurement of the outcome    Were outcome assessors blind to treatment assignment?      Outcome 1:  Outcome 2:  Outcome 3:  Outcome 4:  Outcome 5:  Outcome 6:      Were outcomes measured in the same way for treatment groups?        Outcome 1:  Outcome 2:  Outcome 3:  Outcome 4:  Outcome 5:  Outcome 6:      Were outcomes measured in a reliable way?    Outcome 1:  Outcome 2:  Outcome 3:  Outcome 4:  Outcome 5:  Outcome 6: |  | Yes, No, Unclear, N/A  Yes, No, Unclear, N/A  Yes, No, Unclear, N/A  Yes, No, Unclear, N/A  Yes, No, Unclear, N/A  Yes, No, Unclear, N/A                  Yes, No, Unclear, N/A  Yes, No, Unclear, N/A  Yes, No, Unclear, N/A  Yes, No, Unclear, N/A  Yes, No, Unclear, N/A  Yes, No, Unclear, N/A              Yes, No, Unclear, N/A  Yes, No, Unclear, N/A  Yes, No, Unclear, N/A  Yes, No, Unclear, N/A  Yes, No, Unclear, N/A  Yes, No, Unclear, N/A |
| Bias related to participant retention    Was follow-up complete and if not, were differences between groups in terms of their follow-up adequately described and analysed?      Outcome 1:  -Result 1:  -Result 2:  -Result 3:      Outcome 2:  -Result 1:  -Result 2:  -Result 3:      Outcome 3:  -Result 1:  -Result 2:  -Result 3:      Outcome 4:  -Result 1:  -Result 2:  -Result 3:      Outcome 5:  -Result 1:  -Result 2:  -Result 3:      Outcome 6:  -Result 1:  -Result 2:  -Result 3: |  | Yes, No, Unclear, N/A  Yes, No, Unclear, N/A  Yes, No, Unclear, N/A        Yes, No, Unclear, N/A  Yes, No, Unclear, N/A  Yes, No, Unclear, N/A        Yes, No, Unclear, N/A  Yes, No, Unclear, N/A  Yes, No, Unclear, N/A        Yes, No, Unclear, N/A  Yes, No, Unclear, N/A  Yes, No, Unclear, N/A        Yes, No, Unclear, N/A  Yes, No, Unclear, N/A  Yes, No, Unclear, N/A        Yes, No, Unclear, N/A  Yes, No, Unclear, N/A  Yes, No, Unclear, N/A |
| **Statistical Conclusion Validity** |  |  |
| Were participants analysed in the groups to which they were randomized?    Outcome 1:  -Result 1:  -Result 2:  -Result 3:      Outcome 2:  -Result 1:  -Result 2:  -Result 3:      Outcome 3:  -Result 1:  -Result 2:  -Result 3:      Outcome 4:  -Result 1:  -Result 2:  -Result 3:      Outcome 5:  -Result 1:  -Result 2:  -Result 3:      Outcome 6:  -Result 1:  -Result 2:  -Result 3: |  | Yes, No, Unclear, N/A  Yes, No, Unclear, N/A  Yes, No, Unclear, N/A        Yes, No, Unclear, N/A  Yes, No, Unclear, N/A  Yes, No, Unclear, N/A        Yes, No, Unclear, N/A  Yes, No, Unclear, N/A  Yes, No, Unclear, N/A        Yes, No, Unclear, N/A  Yes, No, Unclear, N/A  Yes, No, Unclear, N/A        Yes, No, Unclear, N/A  Yes, No, Unclear, N/A  Yes, No, Unclear, N/A        Yes, No, Unclear, N/A  Yes, No, Unclear, N/A  Yes, No, Unclear, N/A |
| Was appropriate statistical analysis used?      Outcome 1:  -Result 1:  -Result 2:  -Result 3:      Outcome 2:  -Result 1:  -Result 2:  -Result 3:      Outcome 3:  -Result 1:  -Result 2:  -Result 3:      Outcome 4:  -Result 1:  -Result 2:  -Result 3:      Outcome 5:  -Result 1:  -Result 2:  -Result 3:      Outcome 6:  -Result 1:  -Result 2:  -Result 3: |  | Yes, No, Unclear, N/A  Yes, No, Unclear, N/A  Yes, No, Unclear, N/A        Yes, No, Unclear, N/A  Yes, No, Unclear, N/A  Yes, No, Unclear, N/A        Yes, No, Unclear, N/A  Yes, No, Unclear, N/A  Yes, No, Unclear, N/A        Yes, No, Unclear, N/A  Yes, No, Unclear, N/A  Yes, No, Unclear, N/A        Yes, No, Unclear, N/A  Yes, No, Unclear, N/A  Yes, No, Unclear, N/A        Yes, No, Unclear, N/A  Yes, No, Unclear, N/A  Yes, No, Unclear, N/A |
| Was the trial design appropriate and any deviations from the standard RCT design (individual randomization, parallel groups) accounted for in the conduct and analysis of the trial? |  | Yes, No, Unclear, NA |
| **Overall Appraisal** |  |  |
